# A Synthetic Archaeal Lipid Analog Enables Quantification of Substrate, Products, and Intermediates in the Tetraether Synthase Reaction

**DOI:** 10.64898/2026.09.04.749484

**Published:** 2026-09-09

**Authors:** Cody T. Lloyd, Bo Wang, Squire J. Booker

## Abstract

Glycerol dibiphytanyl glycerol tetraether (GDGT) and macrocyclic diether lipids (mAG) are unique membrane lipids predominantly found in archaea. Both lipids are composed of the same structural components: polar headgroups, glycerol backbones, and ether-linked 40-carbon biphytanyl chains. Tes, a member of the radical *S* -adenosylmethionine enzyme superfamily, synthesizes the biphytanyl chain by catalyzing carbon-carbon (C-C) bond formation between the terminal carbons of two phytanyl chains of the saturated diether lipid substrate. The coupling between the termini of two phytanyl chains on one glycerophospholipid yields mAG, whereas two couplings between the phytanyl termini of two glycerophospholipids yield GDGT. Quantification of the products and presumed intermediates formed during this multistep reaction has been challenging using mass spectrometry due to the lack of appropriate standards for each species. Herein, we designed and synthesized a diether lipid substrate analog containing a fluorescent/UV-visible tag linked to the headgroup, enabling the quantification of each species formed during a Tes reaction. Importantly, under laboratory conditions at 45 °C, we report that GDGT and mAG are initially formed in a 1:1.65 ratio, which differs considerably from the previously assumed ratio.

## Full Text Availability

The license terms selected by the author(s) for this preprint version do not permit archiving in PMC. The full text is available from the preprint server.

